# Combining a Fusion Inhibitory Peptide Targeting the MERS-CoV S2 Protein HR1 Domain and a Neutralizing Antibody Specific for the S1 Protein Receptor-Binding Domain (RBD) Showed Potent Synergism against Pseudotyped MERS-CoV with or without Mutations in RBD

**DOI:** 10.3390/v11010031

**Published:** 2019-01-06

**Authors:** Cong Wang, Chen Hua, Shuai Xia, Weihua Li, Lu Lu, Shibo Jiang

**Affiliations:** 1 Key Laboratory of Medical Molecular Virology of MOE/MOH, School of Basic Medical Sciences and Shanghai Public Health Clinical Center, Fudan University, Shanghai 200032, China; 16111010068@fudan.edu.cn (C.W.); 16211010047@fudan.edu.cn (C.H.); 15111010053@fudan.edu.cn (S.X.); 2 NHC Key Laboratory of Reproduction Regulation (Shanghai Institute of Planned Parenthood Research), Fudan University, Shanghai 200032, China; weihua.li@sippr.org.cn

**Keywords:** MERS-CoV, RBD, mutation, peptide, neutralizing antibody, combination

## Abstract

Middle East respiratory syndrome coronavirus (MERS-CoV) has continuously posed a threat to public health worldwide, yet no therapeutics or vaccines are currently available to prevent or treat MERS-CoV infection. We previously identified a fusion inhibitory peptide (HR2P-M2) targeting the MERS-CoV S2 protein HR1 domain and a highly potent neutralizing monoclonal antibody (m336) specific to the S1 spike protein receptor-binding domain (RBD). However, m336 was found to have reduced efficacy against MERS-CoV strains with mutations in RBD, and HR2P-M2 showed low potency, thus limiting the clinical application of each when administered separately. However, we herein report that the combination of m336 and HR2P-M2 exhibited potent synergism in inhibiting MERS-CoV S protein-mediated cell–cell fusion and infection by MERS-CoV pseudoviruses with or without mutations in the RBD, resulting in the enhancement of antiviral activity in contrast to either one administered alone. Thus, this combinatorial strategy could be used in clinics for the urgent treatment of MERS-CoV-infected patients.

## 1. Introduction

Middle East respiratory syndrome (MERS) coronavirus (MERS-CoV), a lineage C beta-coronavirus, was reported to cause severe respiratory tract infection [1,2]. To date, 2266 laboratory-confirmed cases of infection with MERS-CoV, including 804 MERS-CoV associated deaths, have been reported to the World Health Organization (WHO) from 27 countries. Currently, no effective therapeutics or vaccines are available to treat or prevent MERS-CoV infection.

The spike (S) protein of MERS-CoV plays important roles in virus attachment, fusion, and entry into the target cell [3,4,5]. Similar to other coronaviruses, the S protein of MERS-CoV consists of S1 and S2 subunits. The S1 subunit is responsible for the binding of the virion by its receptor binding domain (RBD) to the cellular receptor, dipeptidyl peptidase-4 (DPP4), while the S2 subunit mediates the fusion between viral and cellular membranes through the interaction between its HR1 and HR2 domains and entry of the viral genetic materials into the host cell [5,6,7,8]. Thus, both the RBD in S1 subunit and HR1 domain in S2 subunit can serve as important targets for development of antiviral agents against MERS-CoV infection.

Recently, we identified a peptide derived from the HR2 domain of MERS-CoV S protein S2 subunit, designated HR2P, which could interact with the HR1 domain of S protein S2 subunit to form a six-helix bundle (6-HB) complex and block viral fusion and replication with IC_50_s ranging from 0.6 to 1 µM [5]. By replacing amino acid residues at the *i* to *i* + 4 positions with a negatively charged amino acid (e.g., E) and a positively charged amino acid (e.g., K) in HR2P for introduction of intramolecular salt-bridges, the resultant peptide HR2P-M2 exhibited improved solubility, stability, and anti-MERS-CoV activity [5,9]. However, its potency is still not strong enough to warrant clinical development.

By screening an extra-large phage-displayed antibody Fab library, Ying et al. identified a human neutralizing monoclonal antibody (hmAb), m336, which is specific for the RBD in the S protein S1 subunit. It exhibited highly potent neutralizing activity against MERS-CoV infection, both in vitro and in vivo [10,11,12,13]. X-ray crystallography has shown that the binding epitope of m336 on MERS-CoV S protein almost completely overlaps with the binding site of DPP4 [14]. However, the future clinical application of m336 could be limited by its inability to neutralize MERS-CoV strains with mutations in RBD, like the mouse neutralizing mAb Mersmab1 with similar weakness [15,16].

In this study, we compared the sensitivity of a pseudotyped MERS-CoV wild-type strain with that of strains with key mutations, including D509G, D510G, Q522H, and I529T, which were detected in the RBD of some MERS-CoV strains isolated from different regions and at different times throughout the course of the MERS outbreak from 2012 to 2015 [17,18]. We found that these strains with mutations in RBD were significantly less sensitive than the wild-type strain to the neutralizing activity of m336, while the pseudoviruses with or without mutations showed equal sensitivity to the fusion inhibitory activity of HR2P-M2. Interestingly, when m336 was combined with HR2P-M2, a strong synergism emerged against MERS-CoV S-mediated cell–cell fusion and infection by pseudotyped MERS-CoV strains with or without mutations in RBD, suggesting that this combinational therapy could be further developed for clinical use to treat patients infected by the MERS-CoV strains with or without mutations in RBD.

## 2. Materials and Methods 

### 2.1. Cells, Peptides, Human mAb m336, and Plasmids

The 293T cell line was obtained from ATCC (Manassas, VA, USA), and the Huh-7 cell line was from the Cell Bank of the Chinese Academy of Sciences (Shanghai, China). These two cell lines were propagated in Dulbecco’s Modified Eagle’s Medium (DMEM) supplemented with 10% fetal bovine serum (FBS). Peptide HR2P-M2 was synthesized by solid phase peptide synthesis at SYN Inc. (Shanghai, China), and human mAb m336 was provided by Prof. Tianlei Ying at Fudan University, Shanghai, China. Recombinant plasmids encoding the MERS-CoV S protein with D509G, D510G, Q522H, or I529T mutations were kindly provided by Dr. Lanying Du at the New York Blood Center, NY, USA.

### 2.2. Production of Pseudoviruses

MERS-CoV pseudoviruses were constructed as described previously [19,20]. Briefly, 293T cells were plated in a T175 tissue culture flask and incubated at 37 °C for 16 h. Cells were cotransfected with plasmids pNL4-3.luc.RE encoding Env-defective, luciferase-expressing HIV-1 and pcDNA3.1-MERS-CoV-S encoding S protein with or without mutation in RBD at mass ratio of 1:1 using VigoFect (Vigorous Biotechnology, Beijing, China), according to the manufacturer’s recommendation. The supernatant was replaced with fresh DMEM at 8–10 h post-transfection and harvested after incubation for an additional 72 h. In order to remove cell debris, the supernatant was centrifuged at 3000 rpm for 10 min, followed by filtration through a 0.45 µm filter. MERS-CoV pseudovirus in the supernatant was quantified by testing p24 content in the product of MERS-CoV pseudovirus.

### 2.3. Inhibition of Pseudotyped MERS-CoV Infection

A MERS-CoV pseudovirus inhibition assay was performed as previously described [5,15,21]. Briefly, Huh-7 cells were seeded (10^4^ cells/well) into a 96-well plate and incubated overnight at 37 °C. MERS-CoV pseudovirus was incubated with a serially diluted inhibitor for 30 min at 37 °C, followed by the addition of Huh-7 cells. The cells were incubated with or without pseudovirus as virus control and cell control, respectively. The culture was replaced with fresh medium 12 h post-infection and incubated for an additional 72 h. Cells were lysed using lysis reagent (Promega, Madison, WI, USA), and cell lysates were transferred to a 96-well Costar flat-bottom luminometer plate (Corning Costar, New York, NY, USA), followed by the addition of luciferase substrate (Promega) to measure luminescence using an Infinite M200 PRO (Tecan, GröDig, Austria).

### 2.4. Inhibition of MERS-CoV S Protein-Mediated Cell–Cell Fusion

**Figure 1:**
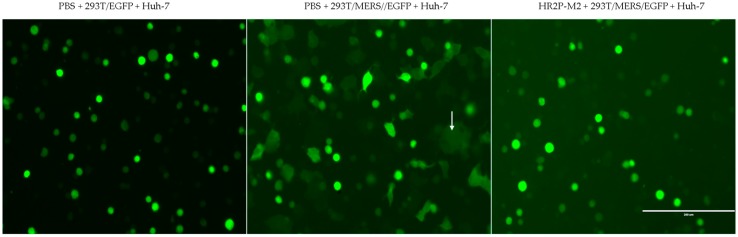
Images of Middle East respiratory syndrome coronavirus (MERS-CoV) S protein-mediated cell–cell fusion. Huh-7 cells were co-cultured with 293T/EGFP cells (**left**) or 293T/MERS/EGFP cells in the presence of PBS (**middle**) or 10 μM HR2P-M2 peptide (**right**) at 37 °C for 2h. The fused cells (one of the fused cell is indicated by an arrow) and the unfused cells were counted under a fluorescence microscope. Scale bars, 200 μm.

MERS-CoV S protein-mediated cell–cell fusion was performed as previously described [5]. Briefly, plasmid pAAV-IRES-MERS-EGFP encoding the MERS-CoV S protein was transfected into 293T cells (293T/MERS/EGFP) using the transfection reagent, VigoFect (Vigorous Biotechnology, Beijing, China). The target Huh-7 cells expressing DPP4were incubated at 2 × 10^4^ cells/well in wells of a 96-well plate for 12 h. The effector 293T/MERS/EGFP cells that express MERS-CoV S protein and EGFP or the control 293T/EGFP cells that express EGFP only were preincubated at 10^4^ cells/well with an inhibitor at the indicated concentration or phosphate buffered saline (PBS) as control at 37 °C for 30 min. The mixture of 293T/MERS/EGFP cells and an inhibitor or PBS were added to Huh-7 cells in the wells, followed by a co-culture at 37 °C for 2 h. The 293T/MERS/EGFP cells fused or unfused with Huh-7 cells were fixed with 4% PFA and counted under an inverted fluorescence microscope (Nikon, Tokyo, Japan). The fused cell showed much larger size and weaker fluorescence intensity than the unfused cell because of the diffusion of EGFP from one cell to more cells (Figure 1). Almost no fused cells could be observed in the groups of negative control (PBS+293T/EGFP+Huh-7) or peptide treatment (HR2P-M2+293T/MERS/EGFP+Huh-7) (Figure 1). The concentration for 50% inhibition (IC_50_) was calculated using CalcuSyn software kindly provided by Dr. T.C. Chou [22].

### 2.5. Inhibitory Activity of Sera from Mice Treated with m336 Alone, HR2P-M2 Alone, or m336/HR2P-M2 Combined

The animal experiment was performed under ethical guidelines for the care and use of laboratory animals of Fudan University, and the protocol was approved by the Institutional Laboratory Animal Care and Use Committee at Fudan University (approval number 20160927-1, 27 September 2016). Six-week-old female specific-pathogen-free (SPF) BALB/c mice (bodyweight about 20 g) were divided into 3 groups of 3 mice each. Mice in group 1, 2, and 3 were intraperitoneally (i.p.) injected with m336 (0.01 mg in 100 µL PBS) alone, HR2P-M2 (1 mg in 100 µL PBS) alone, and the combination of m336 (0.01 mg in 100 µL PBS) and HR2P-M2 (1 mg in 100 µL PBS), respectively. Mice were sedated with Nembutal (100 mg/kg body weight) before and 2 h after injection of the inhibitors, respectively, and bled retro-orbitally. The blood was centrifuged at 6000 rpm for 10 min after standing at room temperature for 3 h. The sera were collected and heat-inactivated at 56 °C for 30 min. Inhibitory activity of the inhibitors on MERS-CoV pseudovirus was evaluated in serum as described above.

### 2.6. Inhibitor Combination Assay 

To assess the potential synergistic effect, HR2P-M2 and m336 were mixed at the indicated molar concentration ratio, while HR2P-M2 alone and m336 alone were included as controls. The mixtures were serially diluted and tested for their inhibitory activity on MERS-CoV pseudovirus infection as described above. Each sample was tested in triplicate, and data were analyzed for synergistic effect by calculating the combination index (CI), using the CalcuSyn program. CI values of <1 and >1 indicate synergy and antagonism, respectively, and synergy was divided into different strengths, according to CI values, as follows: <0.1 indicates very strong synergism; 0.1–0.3 indicates strong synergism; 0.3–0.7 indicates synergism; 0.7–0.85 indicates moderate synergism; and 0.85–0.90 indicates slight synergism [23,24]. Fold of potency enhancement was calculated with the ratio of concentrations of inhibitor testing alone and in combination.

### 2.7. Statistical Analysis

To determine the significance of difference in sensitivity between wild-type and mutant viruses to inhibitors and the inhibitory activity detected in sera from BALB/c mice treated with inhibitors alone or combination, statistical analyses were performed using a two-tailed unpaired Student’s *t*-test, using GraphPad Prism, version 5.0. Values with *p* < 0.05 and *p* < 0.01 were considered statistically significant and very significant, respectively.

## 3. Results

### 3.1. Combining HR2P-M2 with m336 Exhibited Strong Synergism against MERS-CoV Pseudovirus Infection

**Figure 2:**
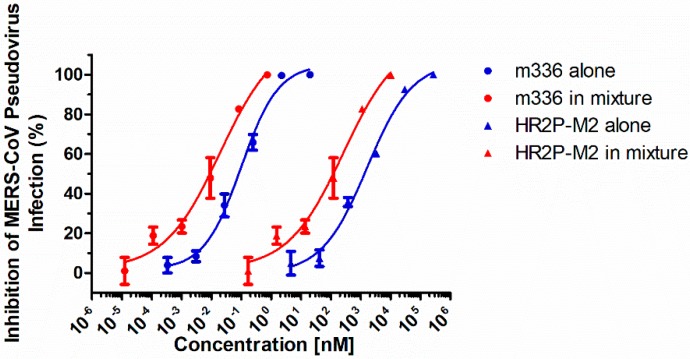
Strong synergism of HR2P-M2 combined with m336 against MERS-CoV pseudovirus infection. The effective concentrations for inhibiting MERS-CoV pseudovirus infection are plotted in two curves. The blue curves represent inhibitors used alone, and the red curves represent each inhibitor used in combination. The width between two curves represents the fold of enhancement between an inhibitor used alone and in combination.

We first investigated the potential cooperative effects of combining HR2P-M2 with m336 on MERS-CoV pseudovirus infection. In our preliminary study, we found that IC_50_ values of HR2P-M2 and m336 for inhibiting MERS-CoV pseudovirus infection were about 600 nM and 0.06 nM, respectively. Therefore, we tested the inhibitory activity of HR2P-M2 alone, m336 alone, and HR2P-M2/m336 in combination at a molar concentration ratio of 10,000:1, respectively. As shown in Figure 2 and Table 1, combining HR2P-M2 and m336 resulted in strong synergistic inhibitory activity against MERS-CoV pseudovirus infection with CI values of 0.13–0.20 for 50–90% inhibition, including potency enhancement of 12.9- to 18.9-fold for m336 and 8.4- to 12.9-fold for HR2P-M2. This result suggested that the MERS-CoV fusion inhibitory peptide HR2P-M2 and the MERS-CoV neutralizing mAb m336 could be used in combination to enhance anti-MERS-CoV activity.

### 3.2. Combining HR2P-M2 with m336 Displayed Strong Synergism against MERS-CoV S Protein-Mediated Cell–Cell Fusion 

**Figure 3:**
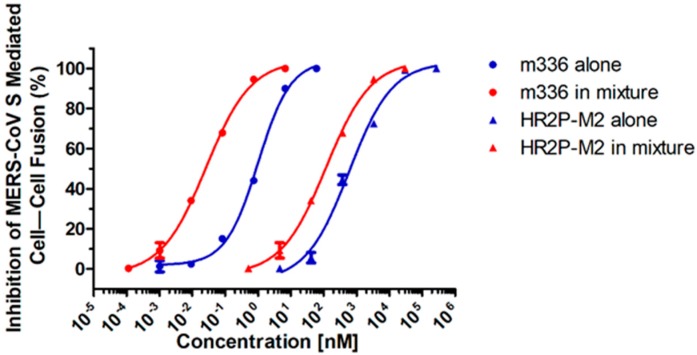
Strong synergism resulting from the HR2P-M2/m336 combination against MERS-CoV S protein-mediated cell–cell fusion.

Next, we tested the potential synergistic activity of the HR2P-M2/m336 combination on MERS-CoV S protein-mediated cell–cell fusion. We adjusted the molar concentration ratio of HR2P-M2 and m336 in the combination to 4500:1, since the IC_50_ values of HR2P-M2 and m336 for inhibiting MERS-CoV S protein-mediated cell–cell fusion in our preliminary studies were about 700 nM and 0.15 nM, respectively. As shown in Figure 3 and Table 2, the combination also exhibited strong synergism against MERS-CoV S protein-mediated cell–cell fusion (CI = 0.27) with enhancement of 18-fold for m336 and 4-fold for HR2P-M2. This result confirms that combining HR2P-M2, a MERS-CoV fusion inhibitor, with m336, a human neutralizing mAb, results in strong synergism on S protein-mediated membrane fusion because they target the different stages of MERS-CoV fusion and entry processes.

### 3.3. MERS-CoV Pseudoviruses with Mutations in RBD Mutant of MERS-CoV Were Resistant to RBD-Specific mAb m336, While They Were Equally Sensitive to the HR1-Targeting Peptide HR2P-M2

Du et al. have previously shown that MERS-CoV pseudoviruses with mutations in RBD, such as D509G and D510G detected in some MERS-CoV strains isolated from different regions and at different times [17,18], are resistant to the neutralizing activity of an RBD-specific mouse mAb Mersmab1 [16]. In the present study, the sensitivity of pseudotyped MERS-CoV strains with key mutations in RBD, as identified in some MERS-CoV mutants isolated during the 2012–2015 outbreaks [17], including D509G, D510G, Q522H, and I529T, along with wild-type MERS-CoV, was compared between the inhibitory activity of HR2P-M2 peptide alone and m336 neutralizing mAb alone. As shown in Table 3, the resistance of MERS-CoV mutants to the neutralizing activity of m336 is about 2- to 8-fold, whereas the pseudoviruses with or without mutations were equally sensitive to fusion inhibitory activity of HR2P-M2. This result suggested that use of mAb m336 alone is unable to control the infection by MERS-CoV strains with mutations in RBD.

### 3.4. Combining m336 with HR2P-M2 Exhibited Potent Synergism against MERS-CoV Pseudoviruses with or without Mutations in RBD or Those in the HR1 Domain

To determine whether the combination of HR2P-M2 and m336 also exhibited synergistic antiviral activity against infection of MERS-CoV strains with mutations in RBD or in the HR1 domain, we constructed pseudoviruses bearing MERS-CoV S protein with mutations in RBD, including D509G, D510G, Q522H, or I529T, and those in the HR1 domain, including Q1020H and Q1020R [25,26]. We then tested their sensitivity to the inhibition of HR2P-M2 alone, m336 alone, and the HR2P-M2/m336 combination. As shown in Table 4, combining m336 with HR2P-M2 exhibited strong synergism against infection by pseudotyped MERS-CoV strains with or without mutations in the RBD or HR1 domain with CI value less than 0.3 and potency enhancement in the range of 6- to 25-fold, suggesting that this combinational therapy has potential to be further developed for treatment of patients infected by different MERS-CoV strains, including those with resistance to RBD-specific neutralizing antibodies.

### 3.5. Sera from Mice Treated with the m336/HR2P-M2 Combination Showed More Efficacy in Inhibiting MERS-CoV Pseudovirus Infection than Either HR2P-M2 or m336 Alone

**Figure 4:**
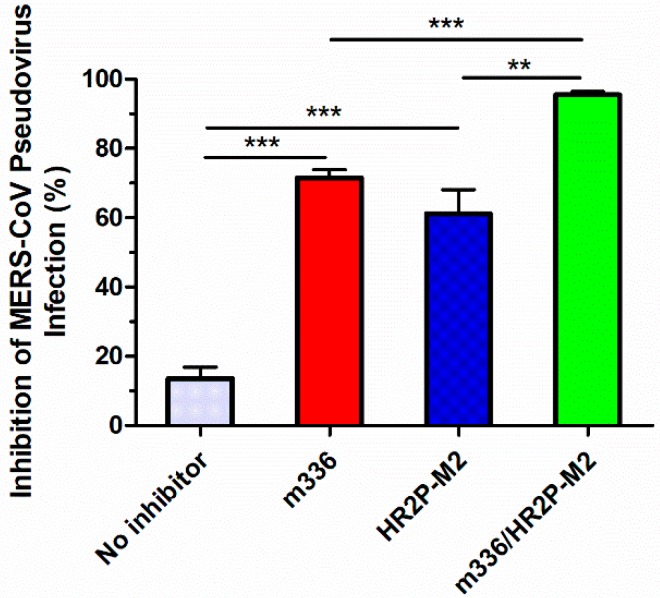
MERS-CoV pseudovirus inhibitory activity as determined from sera of BALB/c mice treated with m336 (0.01 mg) alone, HR2P-M2 (1 mg) alone, or m336 (0.01 mg)/HR2P-M2 (1 mg) in combination. Data are presented as means ± SD. **, and *** represent *p* < 0.01, and *p* < 0.001, respectively.

To determine whether the HR2P-M2/m336 combination could sustain its efficacy in vivo compared to HR2P-M2 or m336 alone, we tested the anti-MERS-CoV pseudovirus activity of the inhibitors in sera of mice treated with i.p. injection of HR2P-M2, m336, and the HR2P-M2/m336 combination, respectively. As shown in Figure 4, the inhibitory activity detected in sera from mice treated with HR2P-M2 or m336 alone was significantly higher than that detected in sera from mice before inhibition of any inhibitor. On the other hand, the anti-MERS-CoV activity detected in sera from mice treated with the HR2P-M2/m336 combination was significantly more potent than that detected in sera of mice administered with HR2P-M2 or m336 alone. This result confirms that combining HR2P-M2 with m336 affords synergism against MERS-CoV S infection, both in vitro and in vivo.

## 4. Discussion

The high mortality of MERS-CoV-infected patients [27,28,29] calls for the development of highly effective anti-MERS-CoV therapeutics. Although we and others have previously identified a MERS-CoV fusion inhibitory peptide (HR2P-M2) targeting the MERS-CoV S2 protein HR1 domain and a highly potent human neutralizing mAb (m336) targeting the MERS-CoV S1 protein RBD [10,26,30], their further development is limited by low potency in the case of HR2P-M2 and low efficacy to neutralize MERS-CoV strains with RBD mutations in the case of m336 [10,31,32,33].

The combinatorial use of drugs with different mechanisms of action, i.e., cocktail regimen, has been widely applied in clinics [22]. For example, the combinatorial use of HIV reverse transcriptase (RT) inhibitors and protease inhibitors, known as highly active anti-retrovirus therapy (HAART), has shown significant synergism in inhibiting HIV-1 infection, reducing adverse effects and delaying the emergence of drug resistance, thus extending the lifespan of millions of HIV/AIDS patients [34,35,36]. Moreover, we previously showed that combining HIV-1 attachment inhibitors with RT inhibitors, or combining the 1st, 2nd, and/or 3rd generation HIV fusion inhibitors that target different sites in the HIV-1 gp41 HR1 domain, exhibited synergistic and complementary effect against infection by a broad spectrum of HIV-1 strains, including those resistant to HIV attachment inhibitors, fusion inhibitors, and RT inhibitors [37,38,39].

In this study, we compared the anti-MERS-CoV activity of HR2P-M2 alone and m336 alone with that of HR2P-M2/m336 in combination and found that the inhibitory activity of the HR2P-M2/m336 combination was significantly more potent than either one administered alone against MERS-CoV S protein-mediated cell–cell fusion and MERS-CoV pseudovirus infection, suggesting synergistic activity based on the dual mechanisms of action whereby HR2P-M2 targets the S2 subunit HR1 domain for inhibiting S2-mediated virus–cell or cell–cell fusion [5] and m336 targets the S1 subunit RBD for inhibiting virus–cell binding or virus attachment [10]. It has been well known that drug synergism can be expected when drugs that act by different mechanisms of action are mixed together [22]. While MERS-CoV pseudoviruses with mutations in RBD were resistant to the RBD-specific mAb m336, they were equally sensitive to HR1-targeting peptide HR2P-M2. Notably, however, the HR2P-M2/m336 combination exhibited strong synergistic antiviral activity against all pseudotyped MERS-CoV strains, including those with mutations in RBD of S protein, which are even resistant to an RBD-specific mouse mAb Mersmab1 [16]. We also demonstrated that sera from mice treated with the HR2P-M2/m336 combination revealed significant efficacy in inhibiting MERS-CoV pseudovirus infection compared to HR2P-M2 or m336 alone. Collectively, these results suggest that the combinatorial strategy overcomes the weaknesses of HR2P-M2 peptide and m336 mAb, while, at the same time, takes advantage of the unique mechanism of action of each to provide, by the sum of both, much more effective inhibitory activity against MERS-CoV infection than either peptide or mAb used alone. The strong synergy of the combination is expected to reduce the dosage of the individual inhibitor in such combinational therapy, resulting in decreased cost and toxicity, thus making the final product more affordable and safer. Therefore, this combinational therapy shows promise for further clinical development.

## Figures and Tables

**Table 1:** Combination index (CI) and fold of enhancement for inhibiting MERS-CoV pseudovirus infection by HR2P-M2 (μM) and m336 (nM) tested in combination.

% Inhibition	CI	HR2P-M2			m336		
		Concentration (μM)		Fold of	Concentration (nM)		Fold of
		Alone	in Mixture	Enhancement	Alone	in Mixture	Enhancement
50	0.197	0.574	0.069	8.36	0.066	0.005	12.94
60	0.183	0.874	0.097	9.05	0.099	0.007	13.88
70	0.168	1.381	0.140	9.87	0.155	0.010	14.98
80	0.152	2.415	0.220	10.96	0.268	0.016	16.44
90	0.131	5.598	0.436	12.85	0.610	0.032	18.92

Note: Each sample was tested in triplicate, and the mean values are presented. Ratio of molar concentration of HR2P-M2 and m336 in combination is 10,000:1.

**Table 2:** Combination index and fold of enhancement for inhibiting MERS-CoV S protein-mediated cell–cell fusion by the HR2P-M2/m336 combination.

% Inhibition	CI	HR2P-M2			m336		
		Concentration (μM)		Fold of	Concentration (nM)		Fold of
		Alone	in Mixture	Enhancement	Alone	in Mixture	Enhancement
50	0.271	0.511	0.110	4.64	0.440	0.024	17.96
60	0.274	0.713	0.156	4.57	0.625	0.035	18.04
70	0.278	1.025	0.228	4.50	0.918	0.051	18.12
80	0.282	1.596	0.362	4.41	1.466	0.080	18.22
90	0.288	3.106	0.726	4.28	2.965	0.161	18.38

Note: Each sample was tested in triplicate, and the mean values are presented. The molar concentration ratio of HR2P-M2 and m336 in combination is 4500:1.

**Table 3:** Sensitivity of MERS-CoV pseudoviruses with or without mutations in the receptor-binding domain (RBD) to the inhibitory activity of m336 (nM) and HR2P-M2 (μM) separately.

MERS-CoV Pseudovirus	IC_50_ (nM) of m336	RR_50_ (Fold of Resistance)	*p*	IC_50_ (μM) of HR2P-M2	RR_50_ (Fold of Resistance)	*p*
Wild-type	0.055 ± 0.009	—	—	0.553 ± 0.056	—	—
D509G	0.116 ± 0.020	2.11	<0.01	0.619 ± 0.079	1.12	>0.05
D510G	0.450 ± 0.085	8.18	<0.05	0.679 ± 0.144	1.23	>0.05
Q522H	0.148 ± 0.051	2.69	<0.01	0.677 ± 0.071	1.22	>0.05
I529T	0.215 ± 0.055	3.91	<0.01	0.574 ± 0.209	1.04	>0.05

Note: Each sample was tested in triplicate. Data are presented as means ± SD. Resistance ratio (RR_50_) values are based on IC_50_ of a mutant strain divided by IC_50_ of the wild-type stain. The significance of the difference between a mutant strain and the wild-type strain was statistically analyzed by a two-tailed unpaired Student’s *t*-test using GraphPad Prism, version 5.0. Values with *p* < 0.05 and *p* < 0.01 were considered statistically significant and very significant, respectively.

**Table 4:** Combination index and fold of enhancement for inhibiting MERS-CoV pseudoviruses with or without mutations in RBD in S1 subunit and HR1 in S2 subunit of MERS-CoV S protein by HR2P-M2 and m336.

% Inhibition	CI	HR2P-M2			m336		
		Concentration (μM)		Fold of	Concentration (μM)		Fold of
		Alone	in Mixture	Enhancement	Alone	in Mixture	Enhancement
Wild type							
50	0.197	0.574	0.069	8.36	0.066	0.005	12.94
60	0.183	0.874	0.097	9.05	0.099	0.007	13.88
70	0.168	1.381	0.140	9.87	0.155	0.010	14.98
80	0.152	2.415	0.220	10.96	0.268	0.016	16.44
90	0.131	5.598	0.436	12.85	0.610	0.032	18.92
D509G in RBD							
50	0.296	0.912	0.155	5.88	0.273	0.034	7.91
60	0.29	1.335	0.217	6.15	0.379	0.048	7.87
70	0.282	2.023	0.313	6.47	0.544	0.070	7.83
80	0.274	3.358	0.489	6.87	0.844	0.109	7.77
90	0.263	7.199	0.956	7.53	1.635	0.213	7.69
D510G in RBD							
50	0.137	0.962	0.088	10.96	0.429	0.020	21.96
60	0.145	1.523	0.153	9.96	0.763	0.034	22.43
70	0.155	2.512	0.280	8.97	1.429	0.062	22.97
80	0.169	4.625	0.586	7.90	3.075	0.130	23.63
90	0.194	11.59	1.777	6.52	9.74	0.395	24.67
Q522H in RBD							
50	0.135	0.799	0.045	17.59	0.129	0.010	12.76
60	0.143	1.243	0.067	18.67	0.166	0.015	11.21
70	0.153	2.012	0.101	19.93	0.219	0.022	9.74
80	0.168	3.619	0.168	21.57	0.306	0.037	8.21
90	0.199	8.757	0.36	24.30	0.508	0.08	6.34
I529T in RBD							
50	0.256	0.864	0.098	8.81	0.153	0.022	7.03
60	0.242	1.295	0.139	9.32	0.23	0.031	7.45
70	0.227	2.013	0.203	9.90	0.358	0.045	7.92
80	0.211	3.449	0.323	10.67	0.614	0.072	8.55
90	0.188	7.752	0.649	11.94	1.383	0.144	9.58
Q1020H in HR1							
50	0.186	0.762	0.086	8.83	0.088	0.006	13.82
60	0.189	0.954	0.106	9.04	0.100	0.008	12.84
70	0.192	1.218	0.131	9.27	0.115	0.010	11.85
80	0.198	1.642	0.172	9.56	0.137	0.013	10.74
90	0.208	2.571	0.257	10.01	0.176	0.019	9.27
Q1020R in HR1							
50	0.293	0.69	0.119	5.79	0.073	0.009	8.30
60	0.28	0.905	0.143	6.32	0.087	0.011	8.19
70	0.268	1.216	0.175	6.95	0.104	0.013	8.06
80	0.254	1.743	0.223	7.80	0.131	0.017	7.92
90	0.238	2.997	0.323	9.29	0.184	0.024	7.70

Note: The molar concentration ratio of HR2P-M2 and m336 in combination against wildtype virus, viruses with mutations in RBD, and those in HR1 is 10,000:1, 4500:1, and 10,000:1, respectively.
